# Supplementary material for: In or out: Response slowing across housing conditions as a measure of affect in three Western lowland gorillas (Gorilla gorilla gorilla)
Source: PeerJ. 2020 Jul 10;8:e9525. doi: 10.7717/peerj.9525 (PMC7357556; doi:10.7717/peerj.9525)
Supplement: Supplemental Information 3 [file peerj-08-9525-s003.docx]

**Experiment 1 Codes for Data File**

Subject:

1 = Pende

2 = Chip

3 = Kongo

Response - 0 = right, 1 = left, 2 = center

Type – 0 = non-threatening, 1= control, 2 = threatening

Access involves South Day Room and North Day room (not analyzed)

Access_Collapsed: Daytime Conditions

1 = Outside, 2 = Inside, 3 = Accessed

Slept Collapsed : Nightime conditions

1 = aisle

2 = dayroom

3 = access

**Experiment 2 Codes**

Subject:

1 = Pende

2 = Chip

3 = Kongo

Response - 0 = right, 1 = left, 2 = center

Type – 0 = averted gaze, 1= control, 2 = direct gaze

Access involves South Day Room and North Day room (not analyzed)

Access_Collapsed: Daytime Conditions

1 = Outside, 2 = Inside, 3 = Accessed

Slept Collapsed : Nightime conditions

1 = aisle , 2 = dayroom, 3 = access

Night – 0 = stall, 1 = dayroom

Day 0 = dayroom, 1 = access
